# Draft Genome Sequence of *Pseudomonas azotifigens* Strain DSM 17556^T^ (6H33b^T^), a Nitrogen Fixer Strain Isolated from a Compost Pile

**DOI:** 10.1128/genomeA.00893-13

**Published:** 2013-10-31

**Authors:** Antonio Busquets, Arantxa Peña, Margarita Gomila, Magdalena Mulet, Joan Mayol, Elena García-Valdés, Antonio Bennasar, Marcel Huntemann, James Han, I-Min Chen, Konstantinos Mavromatis, Victor Markowitz, Krishnaveni Palaniappan, Natalia Ivanova, Andrew Schaumberg, Amrita Pati, T. B. K. Reddy, Henrik Nordberg, Tanja Woyke, Hans-Peter Klenk, Nikos Kyrpides, Jorge Lalucat

**Affiliations:** Microbiologia, Departament de Biologia, Universitat de les Illes Balears, Palma de Mallorca, Spaina; Institut Mediterrani d’Estudis Avançats (IMEDEA, CSIC-UIB), Universitat de les Illes Balears, Palma de Mallorca, Spainb; Instituto Universitario de Investigaciones en Ciencias de la Salud (IUNICS-UIB), Universitat de les Illes Balears, Palma de Mallorca, Spainc; DOE Joint Genome Institute, Walnut Creek, California, USAd; Leibniz Institute, DSMZ, German Collection of Microorganisms and Cell Cultures, Braunschweig, Germanye

## Abstract

*Pseudomonas azotifigens* strain 6H33b^T^ is a nitrogen fixer isolated from a hyperthermal compost pile in 2005 by Hatayama and collaborators. Here we report the draft genome, which has an estimated size of 5.0 Mb, exhibits an average G+C content of 66.73%, and is predicted to encode 4,536 protein-coding genes and 100 RNA genes.

## GENOME ANNOUNCEMENT

Strain 6H33b^T^ was isolated by Hatayama and collaborators from a hyperthermal compost pile in Japan because of its ability to grow diazotrophically (1). It was proposed as the type and unique strain of *Pseudomonas azotifigens* (1). Phylogenetic analysis indicated that *Pseudomonas indica* (98% identity in 16S rRNA genes) in the *Pseudomonas aeruginosa* group was its closest neighbor. A multilocus sequence analysis of four housekeeping genes later demonstrated that it belongs to the *Pseudomonas stutzeri* phylogenetic group (2). The nitrogen-fixing ability within the genus *Pseudomonas* has been debated for many years (3), and the only strains recognized to fix nitrogen are *P. azotifigens* and some members of *P. stutzeri*.

The whole-genome shotgun sequence of strain 6H33b^T^ (DSM 17556^T^) was obtained in the context of the Genomic Encyclopedia of Type Strains (4). The draft genome of *P. azotifigens* DSM 17556^T^ was generated at the Department of Energy (DOE) Joint Genome Institute (JGI) using the Illumina technology (5). An Illumina standard shotgun library was constructed and sequenced using the Illumina HiSeq 2000 platform, which generated 12,314,136 reads totaling 1,847.1 Mb. Illumina sequencing and library artifacts were removed using Duk filtering (L. Mingkun, A. Copeland, and H. J. Duk, unpublished data). Filtered Illumina reads were assembled using Velvet (version 1.1.04) (6), simulated paired-end reads were created from Velvet contigs using wgsim (https://github.com/lh3/wgsim), and simulated read pairs were reassembled using Allpaths-LG (version r42328) (7), resulting in an assembly of 5.0 Mb in 59 scaffolds (96 contigs) with an average 122.8× coverage of the genome.

Protein-coding genes were identified using Prodigal (8); protein product names were assigned by the DOE-JGI Microbial Annotation Pipeline (9) based on the hits to the TIGRfam, Pfam, KEGG, COG, and InterPro databases. Noncoding RNAs were identified using the DOE-JGI Microbial Annotation Pipeline (9). Additional gene prediction analysis and manual functional annotation were performed within the Integrated Microbial Genomes (IMG) platform (10). The G+C mole percent is 66.73%, with 4,636 genes (4,536 protein-coding genes) with function prediction for 3,477 of them. A total of 100 RNA genes were detected. A complete set of nitrogen-fixation genes was found, as well as other genes characteristic for the genus and species (details are given in the IMG database) (10).

Whole-genome sequences of 14 *P. stutzeri* strains are publicly available, and 5 of them are considered nitrogen fixers (11–19). Genome analysis confirmed that *P. azotifigens* exhibited overall similarity to the previously sequenced *P. stutzeri* strains of genomovars 1, 2, 3, 8, and 19. *P. azotifigens* showed ANIb values (20) of 77.67% to 80.57% with *P. stutzeri* strains, which demonstrate the close genetic relationship between both species. Together with phenotypic properties, the absence of denitrification genes and the high GC content confirmed the previous proposal of Hatayama and collaborators that *P. azotifigens* represents a distinct species (1). Comparative genomics of *P. azotifigens* and *P. stutzeri* will facilitate the understanding of the phylogeny of the *nif* operon in *Pseudomonas* species and related genera.

### Nucleotide sequence accession numbers. 

This whole-genome shotgun project has been deposited at DDBJ/EMBL/GenBank under the accession number AUDU00000000. The version described in this paper is the first version, AUDU01000000.
